# Age-Normalized Baseline Renal Function: A Simple Risk Marker in Coronary Artery Bypass Grafting

**DOI:** 10.3390/biomedicines14102386

**Published:** 2026-10-19

**Authors:** Zinar Apaydın, Barış Timur, Mervan Akyol, Salih Güler, İsa Can, Ersin Kadiroğulları

**Affiliations:** Department of Cardiovascular Surgery, Mehmet Akif Ersoy Thoracic and Cardiovascular Surgery Training and Research Hospital, Istanbul 34303, Türkiye; dr.baristimur@gmail.com (B.T.); drmervanakyol@gmail.com (M.A.); drsalihguler@gmail.com (S.G.); iscan3181@gmail.com (İ.C.); ersinkadirogullari@gmail.com (E.K.)

**Keywords:** coronary artery bypass grafting, glomerular filtration rate, preoperative risk stratification, eGFR-to-Age ratio, postoperative mortality

## Abstract

**Objective:** Creatinine-based estimated glomerular filtration rate (eGFR) equations incorporate age but do not normalize renal function to age-expected values. We evaluated whether the eGFR-to-Age ratio is associated with outcomes after isolated coronary artery bypass grafting (CABG). **Methods:** This single-center retrospective cohort included 1311 patients. Multivariable and Firth penalized logistic regression, DeLong receiver operating characteristic (ROC) comparisons, bootstrap internal validation, and outcome-specific Youden cut-offs were used. **Results:** Thirty-day mortality occurred in 19 (1.4%) and postoperative dialysis in 17 (1.3%) patients. After adjustment, each 0.1-unit increase in the ratio was associated with lower odds of mortality, with odds ratio (OR) 0.786, 95%; confidence interval (CI) 0.679–0.915, and dialysis (OR 0.735, 95% CI 0.633–0.851). For mortality, discrimination of the ratio (AUC 0.818) was comparable to that of eGFR and age entered separately (AUC 0.831, *p* = 0.195) and higher than that of eGFR alone (AUC 0.767, *p* = 0.005) and European System for Cardiac Operative Risk Evaluation (EuroSCORE) II (AUC 0.722, *p* = 0.018). Negative predictive values exceeded 99%, largely reflecting low event rates, whereas positive predictive values were below 5%. **Conclusions:** The eGFR-to-Age ratio is a simple single-parameter summary of eGFR and age, with discrimination comparable to that of its two components. It may serve as a supplementary bedside screening parameter rather than a replacement for comprehensive risk scores, pending external validation.

## 1. Introduction

Coronary artery bypass grafting (CABG) remains the gold standard revascularization strategy for multivessel and left main coronary artery disease, offering proven long-term survival benefits over percutaneous interventions [1]. Despite advances in surgical technique and perioperative management, postoperative complications remain major sources of morbidity, underscoring the need for accurate preoperative risk stratification [2,3].

EuroSCORE (European System for Cardiac Operative Risk Evaluation) II is the most widely used risk stratification tool in cardiac surgery [4]; however, its performance varies across populations, with a tendency to underestimate operative risk in higher-risk and non-European cohorts [5], highlighting the need for supplementary bedside parameters.

Preoperative renal function is an established independent predictor of adverse outcomes after CABG. Reduced estimated glomerular filtration rate (eGFR) has been consistently associated with postoperative acute kidney injury (AKI), dialysis, prolonged mechanical ventilation, and mortality [6,7,8]. Renal function declines progressively with age, and a given eGFR carries markedly different prognostic implications depending on the patient’s age, a dimension neither eGFR nor age alone captures. Although creatinine-based eGFR equations incorporate age to adjust for muscle mass, this does not normalize renal function relative to age-expected values, leaving age-normalized renal function unaddressed. Patients with normal creatinine but reduced eGFR represent a high-risk group frequently under identified by conventional screening [9], suggesting that standardizing renal function relative to age may provide superior prognostic information.

To our knowledge, the eGFR-to-Age ratio has not been systematically evaluated as a preoperative risk stratification tool in CABG. We investigated whether this ratio is associated with 30-day mortality, postoperative dialysis, and prolonged mechanical ventilation after adjustment for clinical covariates in a large single-center CABG cohort, and compared its discriminatory performance against eGFR alone, age alone, and EuroSCORE II. The intended use of the ratio is as a simple preoperative screening parameter that complements, rather than replaces, comprehensive risk scores.

## 2. Materials and Methods

### 2.1. Study Design and Patient Population

This single-center retrospective cohort study included consecutive adults undergoing isolated CABG at a tertiary cardiovascular surgery referral center between February 2025 and February 2026. Patients undergoing concomitant cardiac procedures (e.g., valve surgery) were excluded to maintain cohort homogeneity. We additionally excluded patients operated under emergency or salvage conditions and those undergoing redo CABG and patients with missing data for any variable required for the primary analysis. The final cohort comprised 1311 patients. This retrospective study was conducted in accordance with the Declaration of Helsinki. All patients provided written informed consent preoperatively for the anonymized use of their clinical data for scientific research. This retrospective study used routinely collected clinical data; no additional tests, procedures, or interventions were performed for research purposes.

### 2.2. Outcome Definitions

Postoperative dialysis was defined as new-onset renal replacement therapy after surgery; none of the three patients receiving preoperative dialysis were classified as having postoperative dialysis. Acute kidney injury was defined according to the Kidney Disease: Improving Global Outcomes (KDIGO) serum creatinine criteria (an increase in serum creatinine of ≥0.3 mg/dL within 48 h or to ≥1.5 times baseline within 7 days [10]); urine output criteria were not applied, and AKI was recorded as a binary variable without staging. Prolonged mechanical ventilation was defined as mechanical ventilation for >24 h after surgery. AKI was analyzed as an additional secondary outcome.

### 2.3. Statistical Analysis

Continuous variables were expressed as median (IQR) and categorical variables as frequency and percentage. Group comparisons used the Mann–Whitney U test and Pearson’s chi-squared test, or Fisher’s exact test when any expected cell count was <5, as appropriate.

Preoperative eGFR was calculated from preoperative serum creatinine using the race-free 2021 Chronic Kidney Disease Epidemiology Collaboration (CKD-EPI) creatinine equation [11]. Serum creatinine measurements were IDMS-standardized. The eGFR-to-Age ratio was analyzed as a continuous variable and dichotomized at <1.135 versus ≥1.135 for the group comparisons in Table 1. This three-decimal threshold was derived from the ROC analysis for 30-day mortality. For the diagnostic performance reported in Section 3.5 outcome-specific Youden cut-offs were used: 1.1355 for 30-day mortality and postoperative dialysis, and 1.5110 for prolonged mechanical ventilation. In summary, the Youden-optimal eGFR-to-Age ratio cut-off for both 30-day mortality and postoperative dialysis was an observed ratio value of 1.1354995, reported as 1.1355 (four decimals) for the diagnostic performance analysis (Section 3.5) and Figure 1 and as 1.135 (three decimals) to define the Low and High groups (Table 1 and Table 2, Figure 2); 1.5110 was the Youden-optimal cut-off for prolonged mechanical ventilation (Section 3.5).

Univariable logistic regression assessed associations between the eGFR-to-Age ratio and binary outcomes; linear regression was used for continuous outcomes. Multivariable models incorporated covariates selected a priori based on clinical relevance and the events-per-variable principle. Given limited outcome events (mortality n = 19, dialysis n = 17), models were restricted to four preoperative covariates. This corresponded to approximately 4.8 and 4.3 events per variable for 30-day mortality and postoperative dialysis, respectively, which is below the conventional threshold of 10; therefore, Firth’s penalized logistic regression was performed as a sensitivity analysis. AKI (276 events) was modeled with the same four preoperative covariates as the primary models, after exclusion of the three patients receiving preoperative dialysis. A sensitivity analysis of postoperative dialysis excluding these three patients was also performed. Because the association between the ratio and AKI departed from linearity, the AKI model was additionally fitted with a restricted cubic spline (4 knots) and with a piecewise linear term (knot at 1.2, chosen post hoc on the basis of observed event rates). Odds ratios for the eGFR-to-Age ratio are reported per 0.1-unit increase. Prolonged mechanical ventilation was pre-specified as a secondary exploratory outcome; its model additionally included CPB time. Age was included in all models to assess the independent contribution of the eGFR-to-Age ratio. Due to high collinearity (VIF > 10), eGFR and eGFR-to-Age ratio were not entered simultaneously. Multicollinearity was assessed using the VIF for all covariates in each multivariable model. Continuous covariates were entered as linear terms; the linearity assumption was assessed for each continuous covariate within its multivariable model by comparing a restricted cubic spline (4 knots; 3 knots for ejection fraction because of tied values) with the linear term using a likelihood-ratio test. Because age is the denominator of the eGFR-to-Age ratio, a sensitivity analysis was performed in which the ratio was replaced by eGFR and age entered as separate continuous terms, with all other covariates unchanged.

Discriminatory performance was assessed by AUC, with pairwise comparisons using the DeLong method. Goodness-of-fit was evaluated using the Hosmer–Lemeshow test. Non-linearity was explored using restricted cubic splines with 4 knots. Internal validation used bootstrap resampling (1000 iterations) with optimism-corrected AUC values reported. Clinical utility was assessed by decision curve analysis and calibration plots. Calibration was additionally summarized by the bootstrap optimism-corrected calibration slope and calibration-in-the-large intercept. To examine the chosen functional form, the ratio was compared with the deviation of observed eGFR from age-expected eGFR (observed eGFR minus eGFR predicted from age by linear regression in this cohort) and with a model including eGFR, age, and their interaction. Because several outcomes and subgroups were analyzed, no correction for multiple comparisons was applied, and secondary outcome and subgroup analyses were considered exploratory. This study is reported in accordance with the TRIPOD statement (checklist in the Supplementary Materials).

All analyses were performed in R version 4.3 using pROC, rms, ResourceSelection, dcurves, ggplot2, gtsummary, and flextable. A two-sided *p* < 0.05 was considered statistically significant.

## 3. Results

### 3.1. Baseline Characteristics

A total of 1311 patients undergoing isolated CABG were included. Median age was 61.0 years (IQR 54.0–67.0), 80.3% were male, and the most common comorbidities were hypertension (61.3%), hyperlipidemia (55.8%), and diabetes mellitus (45.7%). Median preoperative eGFR was 87.53 mL/min/1.73 m^2^ (IQR 73.03–99.53), the median eGFR-to-Age ratio was 1.44 (IQR 1.13–1.76), and median EuroSCORE II was 0.90% (IQR 0.66–1.28) (Table 1).

Thirty-day mortality occurred in 19 patients (1.4%), dialysis in 17 (1.3%), prolonged mechanical ventilation in 295 (22.5%), acute kidney injury in 276 (21.1%), atrial fibrillation in 245 (18.7%), and reintubation in 61 (4.7%).

### 3.2. Group Comparison by eGFR-to-Age Ratio

Using the rounded mortality-derived threshold of 1.135 for group comparisons, 333 patients (25.4%) were classified as the Low eGFR/Age group and 978 (74.6%) as the High eGFR/Age group. Compared with the High group, the Low group was older, more often female, had more hypertension, lower preoperative eGFR, higher EuroSCORE II, and less hyperlipidemia (all *p* < 0.001; Table 1).

Clinically, the Low group had higher rates of 30-day mortality, postoperative dialysis, prolonged mechanical ventilation, and acute kidney injury, together with longer ICU and hospital stays (all *p* < 0.001; Table 2; Supplementary Figure S1). The direction of the association with 30-day mortality was consistent across sex, diabetes, hypertension, and age subgroups, although the confidence interval crossed 1 in patients younger than 65 years (4 events) (Supplementary Figure S2); these subgroup analyses were exploratory and were not adjusted for multiple comparisons.

### 3.3. Univariable and Multivariable Regression Analyses

Univariable analysis confirmed significant associations between the eGFR-to-Age ratio and all outcomes (Supplementary Table S1). Univariable associations of all candidate predictors and the full multivariable model equations, including intercepts, are provided in Supplementary Tables S7 and S8. In multivariable analysis, the ratio remained associated with 30-day mortality (OR 0.786 per 0.1-unit increase, 95% CI 0.679–0.915, *p* = 0.001) and postoperative dialysis (OR 0.735, 95% CI 0.633–0.851, *p* < 0.001) after adjustment for age, ejection fraction, and LIMA use. Age did not retain independent significance in either model (*p* = 0.187 and *p* = 0.936); however, because age is also the denominator of the ratio, this finding partly reflects the variance shared by the two terms and should not be interpreted as an absence of an age effect. LIMA use was independently protective for both outcomes (OR 0.215, *p* = 0.005 and OR 0.227, *p* = 0.014). Model AUC values were 0.858 and 0.869 for mortality and dialysis. For prolonged mechanical ventilation, analyzed as a secondary exploratory outcome, the ratio retained borderline significance (OR 0.953, 95% CI 0.911–0.998, *p* = 0.041); unlike the primary models, age also retained independent significance (*p* = 0.037). Exploratory model AUC was 0.676. Acute kidney injury occurred in 276 of 1308 patients without preoperative dialysis (21.1%). After adjustment, the ratio was associated with AKI (OR 0.822 per 0.1-unit increase, 95% CI 0.783–0.863, *p* < 0.001; model AUC 0.691). In this model, age showed an inverse association with AKI (OR 0.974, 95% CI 0.951–0.998), although its univariable association was positive (OR 1.050 per year; Supplementary Table S7); as in the mortality and dialysis models, this most likely reflects the variance shared by age and the ratio, of which age is the denominator, and should not be interpreted as a protective effect of age. Results are presented in Table 3. The wide confidence intervals around the odds ratios for 30-day mortality and postoperative dialysis reflect the small number of outcome events and indicate considerable statistical imprecision; these estimates should therefore be interpreted with caution. All VIFs were below 3 (eGFR-to-Age ratio 2.84–2.92, age 2.84–2.94, other covariates ≤1.04). No significant departure from linearity was observed for any continuous covariate in the mortality and dialysis models (all *p* > 0.10), whereas departures from linearity were observed for age, albumin, and the eGFR-to-Age ratio in the exploratory prolonged mechanical ventilation model (Supplementary Table S2); in the AKI model, departures from linearity were observed for the eGFR-to-Age ratio and age, with observed AKI rates plateauing at ratios above approximately 1.2 (Supplementary Tables S2 and S4). In a sensitivity analysis, modeling the ratio with a restricted cubic spline improved the fit of the AKI model (likelihood-ratio *p* < 0.001) without changing discrimination (AUC 0.691 vs. 0.691); in a piecewise model, the OR per 0.1-unit increase was 0.679 (95% CI 0.619–0.743) below a ratio of 1.2 and 0.925 (95% CI 0.867–0.986) above 1.2 (Supplementary Table S9). The linear OR for AKI should therefore be interpreted as an average association. In the sensitivity analysis with eGFR and age entered as separate continuous terms, lower eGFR was independently associated with 30-day mortality (OR 0.965 per mL/min/1.73 m^2^, 95% CI 0.943–0.988, *p* = 0.002) and postoperative dialysis (OR 0.954, 95% CI 0.931–0.977, *p* < 0.001); age was independently associated with 30-day mortality (OR 1.090 per year, 95% CI 1.023–1.168, *p* = 0.010) but not with postoperative dialysis (OR 1.050, 95% CI 0.986–1.124, *p* = 0.141). The AUCs of these models (0.855 and 0.864) were similar to those of the corresponding eGFR-to-Age ratio models (0.858 and 0.869) (Supplementary Table S3). In Firth’s penalized regression, the ORs for the ratio were 0.789 (95% CI 0.684–0.915, *p* = 0.002) for 30-day mortality and 0.740 (95% CI 0.641–0.854, *p* < 0.001) for postoperative dialysis. After exclusion of the three patients receiving preoperative dialysis, none of whom were classified as undergoing postoperative dialysis, the association between the ratio and postoperative dialysis was unchanged (OR 0.715, 95% CI 0.609–0.836, *p* < 0.001; Supplementary Table S6).

### 3.4. Discriminatory Performance and ROC Analysis

The eGFR-to-Age ratio demonstrated good discrimination for 30-day mortality (AUC 0.818, 95% CI 0.726–0.909) and postoperative dialysis (AUC 0.824, 95% CI 0.722–0.926), and modest performance for prolonged MV (AUC 0.620, 95% CI 0.583–0.657; Figure 1). The ratio outperformed eGFR alone for both mortality (AUC 0.767, *p* = 0.005) and dialysis (AUC 0.784, *p* = 0.022), and Euro SCORE II for mortality (AUC 0.722, *p* = 0.018). Discrimination was comparable to a model incorporating eGFR and age separately (mortality AUC 0.831, *p* = 0.195; dialysis AUC 0.822, *p* = 0.480), indicating that the ratio summarizes both variables within a single parameter. For AKI, the ratio (AUC 0.686, 95% CI 0.648–0.724) performed similarly to eGFR alone (AUC 0.689, *p* = 0.705) and eGFR plus age (AUC 0.690, *p* = 0.436) and better than Euro SCORE II (AUC 0.645, *p* = 0.016). Compared with alternative functional forms, the deviation of observed from age-expected eGFR discriminated significantly worse than the ratio for 30-day mortality (AUC 0.663, *p* < 0.001) and postoperative dialysis (AUC 0.696, *p* = 0.005), whereas a model including eGFR, age, and their interaction performed similarly to the ratio for all outcomes (Supplementary Table S5). Full comparisons are in Table 4.

### 3.5. Diagnostic Performance at Outcome-Specific Cut-Offs

At the outcome-specific Youden cut-off (eGFR-to-Age ratio 1.1355), the eGFR-to-Age ratio demonstrated a sensitivity of 84.2% (95% CI 59.5–95.8%) and specificity of 75.1% (95% CI 72.6–77.4%) for 30-day mortality, with a negative predictive value of 99.7% (95% CI 99.0–99.9%) and positive likelihood ratio of 3.38. For postoperative dialysis, sensitivity was 88.2% (95% CI 62.3–97.9%), specificity 75.0% (95% CI 72.6–77.4%), and NPV 99.8% (95% CI 99.2–100%). The corresponding positive predictive values were 4.7% (95% CI 2.8–7.7%) for 30-day mortality and 4.4% (95% CI 2.6–7.4%) for postoperative dialysis. Because the cut-off was derived and evaluated in the same cohort, these estimates are likely optimistic, and the high negative predictive values largely reflect the low prevalence of the outcomes (1.4% and 1.3%). To facilitate clinical interpretation, observed event rates across ratio bands and model-predicted probabilities at representative ratio values are provided in Supplementary Table S4; for example, the predicted probability of 30-day mortality was 4.2% at a ratio of 0.8, 1.3% at 1.2, and 0.4% at 1.6. Diagnostic performance is presented in Table 5.

### 3.6. Restricted Cubic Spline Analysis

Restricted cubic spline analysis with 4 knots revealed a monotonically decreasing relationship between the eGFR-to-Age ratio and the predicted probability of 30-day mortality and postoperative dialysis, with non-linearity tests showing no significant departure from linearity for mortality (*p* = 0.980) or dialysis (*p* = 0.429). A modest degree of non-linearity was observed for prolonged mechanical ventilation (*p* = 0.039), with the predicted probability plateauing at higher eGFR-to-Age ratio values. Spline curves with 95% confidence intervals are presented in Figure 2.

### 3.7. Internal Validation

Bootstrap resampling with 1000 iterations demonstrated minimal optimism across all models. Optimism-corrected AUC values were 0.845 for 30-day mortality (apparent AUC 0.858, optimism 0.013), 0.855 for postoperative dialysis (apparent AUC 0.869, optimism 0.014), and 0.669 for prolonged mechanical ventilation (apparent AUC 0.676, optimism 0.007), indicating that the models were not substantially overfitted. For AKI, the optimism-corrected AUC was 0.686 (apparent AUC 0.691, optimism 0.005). Optimism-corrected calibration slopes were 0.920, 0.916, 0.961, and 0.975, and calibration-in-the-large intercepts were 0.028, 0.044, 0.001, and −0.002 for 30-day mortality, postoperative dialysis, prolonged mechanical ventilation, and AKI, respectively. Calibration was adequate for 30-day mortality (Hosmer–Lemeshow *p* = 0.149) and postoperative dialysis (*p* = 0.135), whereas the Hosmer–Lemeshow test indicated significant miscalibration for prolonged mechanical ventilation (*p* = 0.032); predicted probabilities from this exploratory model should therefore be interpreted with caution. Calibration plots are presented in Supplementary Figure S3. The optimism-corrected AUC values are reported above, and the Hosmer–Lemeshow results are presented in Table 5.

### 3.8. Decision Curve Analysis

Decision curve analysis showed net benefit of the eGFR-to-Age ratio model over the treat-all and treat-none strategies for predicting 30-day mortality and postoperative dialysis. Its performance was comparable to that of the model including eGFR and age as separate predictors, whereas EuroSCORE II showed lower net benefit across the evaluated threshold range. Decision curves are presented in Figure 3.

## 4. Discussion

This study evaluated the prognostic value of the eGFR-to-Age ratio for 30-day mortality, postoperative dialysis, and prolonged mechanical ventilation in patients undergoing isolated CABG. The ratio showed good discrimination for 30-day mortality and postoperative dialysis, with negative predictive values exceeding 99% at the outcome-specific Youden cut-offs (largely reflecting the low event rates; positive predictive values <5%), and outperformed eGFR alone in pairwise ROC comparisons. To our knowledge, the eGFR-to-Age ratio has not previously been evaluated as a preoperative risk stratification tool in CABG patients; the present study is among the first to systematically assess its prognostic value in this setting.

Reduced eGFR has been consistently associated with increased postoperative mortality, AKI, dialysis requirement, and prolonged mechanical ventilation across large surgical cohorts [6,12]. Importantly, however, serum creatinine, the most widely used bedside marker of renal function, is strongly influenced by muscle mass, which declines progressively with age. As a consequence, elderly patients may harbor significant renal dysfunction despite a normal serum creatinine level, a phenomenon termed occult renal dysfunction [9]. Both Miceli et al. and Marui et al. demonstrated that occult renal dysfunction independently predicts mortality, dialysis, and other adverse outcomes after CABG [13,14], highlighting that serum creatinine alone systematically underestimates renal risk in older patients.

This age–eGFR interaction has a well-established physiological basis. eGFR declines progressively with aging because of nephron loss, reduced renal perfusion, and glomerulosclerosis [15]. Although creatinine-based equations such as CKD-EPI adjust for age, they do not fully account for the differing prognostic implications of a given eGFR across age groups [16]. Thus, a 70-year-old and a 50-year-old with the same eGFR may have substantially different age-normalized renal function. By expressing renal function relative to age, the eGFR-to-Age ratio captures this distinction in a simple bedside parameter.

Whether the ratio simply reflects its two components rather than providing independent prognostic information is a legitimate concern. Our results indicate that the ratio does not carry predictive information beyond its two components but summarizes them in a single parameter. The eGFR-to-Age ratio remained associated with all three outcomes after adjustment for age, whereas age itself was no longer significant for 30-day mortality (*p* = 0.187) or postoperative dialysis (*p* = 0.936). However, because age is the denominator of the ratio, entering both terms in the same model introduces mathematical dependence between them, and the loss of significance of age partly reflects their shared variance rather than an absence of an age effect. Consistent with this, when eGFR and age were entered as separate continuous terms, age remained independently associated with 30-day mortality, and discrimination was similar to that of the ratio-based models (Supplementary Table S3). Moreover, its discrimination was comparable to a model including eGFR and age as separate covariates (DeLong *p* = 0.195 and *p* = 0.480, respectively), while significantly outperforming eGFR alone for mortality (*p* = 0.005) and postoperative dialysis (*p* = 0.022). Thus, the principal advantage of the ratio is simplicity, a single bedside value, rather than incremental predictive performance.

The choice of a ratio also requires justification. The eGFR-to-Age ratio is not a physiologically derived quantity: its unit (mL/min/1.73 m^2^ per year) has no direct physiological meaning, and because the CKD-EPI equation already incorporates age, age contributes to the ratio twice. We selected the ratio pragmatically, as a single value that can be calculated at the bedside and that increases with better renal function and decreases with advancing age. To examine whether a more principled functional form would perform better, we compared it with the deviation of observed eGFR from age-expected eGFR and with a model including eGFR, age, and their interaction. The deviation measure, which by construction is uncorrelated with age, discriminated significantly worse than the ratio for 30-day mortality and postoperative dialysis, whereas the interaction model performed similarly to the ratio for all outcomes (Supplementary Table S5). Thus, within this cohort, neither alternative formulation discriminated better than the ratio, which is the simplest to apply; age-stratified eGFR thresholds were not evaluated.

The discriminatory performance of the eGFR-to-Age ratio for prolonged mechanical ventilation was modest (AUC 0.620) compared with mortality and dialysis (AUC 0.818 and 0.824), reflecting the multifactorial nature of this outcome. Prolonged mechanical ventilation is influenced by pulmonary function, cardiac output, bleeding, neurological status, and perioperative management, many of which are not captured by a single preoperative renal parameter. Accordingly, both the eGFR-to-Age ratio (*p* = 0.041) and age (*p* = 0.037) remained associated with this outcome, suggesting that age contributes prognostic information beyond age-normalized renal function for ventilatory outcomes. The modest calibration of this model further supports cautious interpretation. CPB time was retained only in the exploratory prolonged mechanical ventilation model because of its direct mechanistic relevance, whereas the primary mortality and dialysis models included only preoperative variables.

Diez et al. demonstrated that lower eGFR independently predicted in-hospital mortality and that eGFR varied substantially with age and sex in 6232 cardiac surgery patients [17]. Our findings extend these observations by showing that normalizing eGFR to age improves discrimination over eGFR alone, while providing prognostic performance comparable to separate adjustment for eGFR and age.

Compared with EuroSCORE II, the eGFR-to-Age ratio showed higher discrimination for 30-day mortality (AUC 0.818 vs. 0.722, DeLong *p* = 0.018) but not for postoperative dialysis (AUC 0.824 vs. 0.803, *p* = 0.649). EuroSCORE II incorporates creatinine clearance as a renal variable but does so in a categorical and dichotomized fashion, which inherently loses the prognostic information carried by the continuous gradient of renal function. EuroSCORE II does not account for age-normalized renal function, potentially misclassifying patients with markedly different age-relative renal function and operative risk. The eGFR-to-Age ratio captures this interaction, which may partly explain this difference. However, EuroSCORE II is a composite, multi-organ risk score, whereas the eGFR-to-Age ratio is a single-organ bedside parameter; the ratio should therefore be regarded as a potential supplementary bedside screening parameter rather than a replacement for comprehensive risk scores. These findings, together with previous reports of suboptimal EuroSCORE II calibration in non-European and Turkish cardiac surgery populations [5,7], suggest that the ratio may complement existing preoperative risk assessment in CABG.

Although postoperative dialysis occurred in only 1.3% of patients, it carries major clinical consequences [3]. At the outcome-specific Youden cut-off of 1.1355, the eGFR-to-Age ratio identified postoperative dialysis with 88.2% sensitivity and a 99.8% negative predictive value. However, the positive predictive value was only 4.4%, and the high negative predictive value primarily reflects the low prevalence of postoperative dialysis (1.3%); because the cut-off was derived and tested in the same cohort, these estimates are optimistic and require external validation. In the Table 1 groups defined at 1.135, 99.7% of patients with a ratio ≥1.135 did not require postoperative dialysis, whereas those below the threshold had markedly higher mortality (4.5% vs. 0.4%) and dialysis (4.2% vs. 0.3%), translating to an approximately 10- to 14-fold elevation in risk compared to the high-ratio group, identifying a subgroup that may benefit from intensified perioperative renal protection in line with KDIGO recommendations [10]. Restricted cubic spline analysis demonstrated a predominantly linear inverse association between the ratio and postoperative risk. Bootstrap validation showed minimal optimism, although resampling from so few events may underestimate the true optimism, while decision curve analysis demonstrated consistent net clinical benefit across clinically relevant threshold probabilities [18] (Figure 3).

Several limitations of this study deserve acknowledgment. The retrospective, single-center design limits generalizability, and external validation in independent cohorts is required before the eGFR-to-Age ratio can be recommended for routine clinical use. Moreover, our cohort was a low-risk population (median EuroSCORE II 0.90%, 30-day mortality 1.4%), and emergency, salvage, redo, and combined procedures were excluded; the findings therefore apply only to low-risk patients undergoing non-emergency isolated primary CABG, and the performance of the ratio in higher-risk patients, in whom risk stratification is most needed, remains unknown. The relatively small number of outcome events, 19 deaths and 17 dialysis cases, limits the precision of the estimates, as reflected by the wide confidence intervals of the odds ratios (e.g., OR per 0.1-unit increase 0.786, 95% CI 0.679–0.915 for 30-day mortality; 0.735, 95% CI 0.633–0.851 for postoperative dialysis), and precluded the development of more complex multivariable models, although bootstrap internal validation demonstrated acceptable model stability (bootstrap resampling from a small number of events may nevertheless underestimate the true optimism). Finally, the eGFR-to-Age ratio inherits the known limitations of creatinine-based eGFR estimation, including sensitivity to muscle mass, nutritional status, and acute physiological perturbations; neither cystatin C [16] nor novel tubular injury biomarkers (e.g., NGAL or KIM-1) were available, and these may offer complementary information in future studies. In addition, potential confounders such as preoperative intra-aortic balloon pump use, exposure to nephrotoxic medications (e.g., ACE inhibitors/angiotensin receptor blockers or NSAIDs), and the timing of preoperative contrast administration were not accounted for in our analyses. Furthermore, multiple outcomes and subgroups were analyzed without correction for multiple comparisons; findings for secondary outcomes and subgroups should be considered hypothesis-generating.

## 5. Conclusions

The preoperative eGFR-to-Age ratio is a simple, readily calculable bedside parameter that was associated with 30-day mortality and postoperative dialysis after adjustment in patients undergoing isolated CABG. The ratio shows good discrimination for 30-day mortality and postoperative dialysis, with negative predictive values exceeding 99% at the outcome-specific Youden cut-offs, which largely reflect the low event rates (positive predictive values < 5%), and discriminates better than eGFR alone and, for 30-day mortality, than EuroSCORE II in pairwise comparisons. Its discrimination was comparable to that of eGFR and age entered separately, with no statistically significant difference; its principal advantage is therefore simplicity as a single bedside parameter rather than additional predictive information. The rounded 1.135 threshold used for group comparisons was derived from the mortality ROC analysis and should be considered exploratory pending external validation in independent cohorts. These findings suggest that the eGFR-to-Age ratio may serve as a valuable complement to existing risk stratification tools in the preoperative evaluation of CABG candidates, enabling the identification of high-risk patients who may benefit from targeted perioperative renal protective strategies.

## Figures and Tables

**Figure 1:**
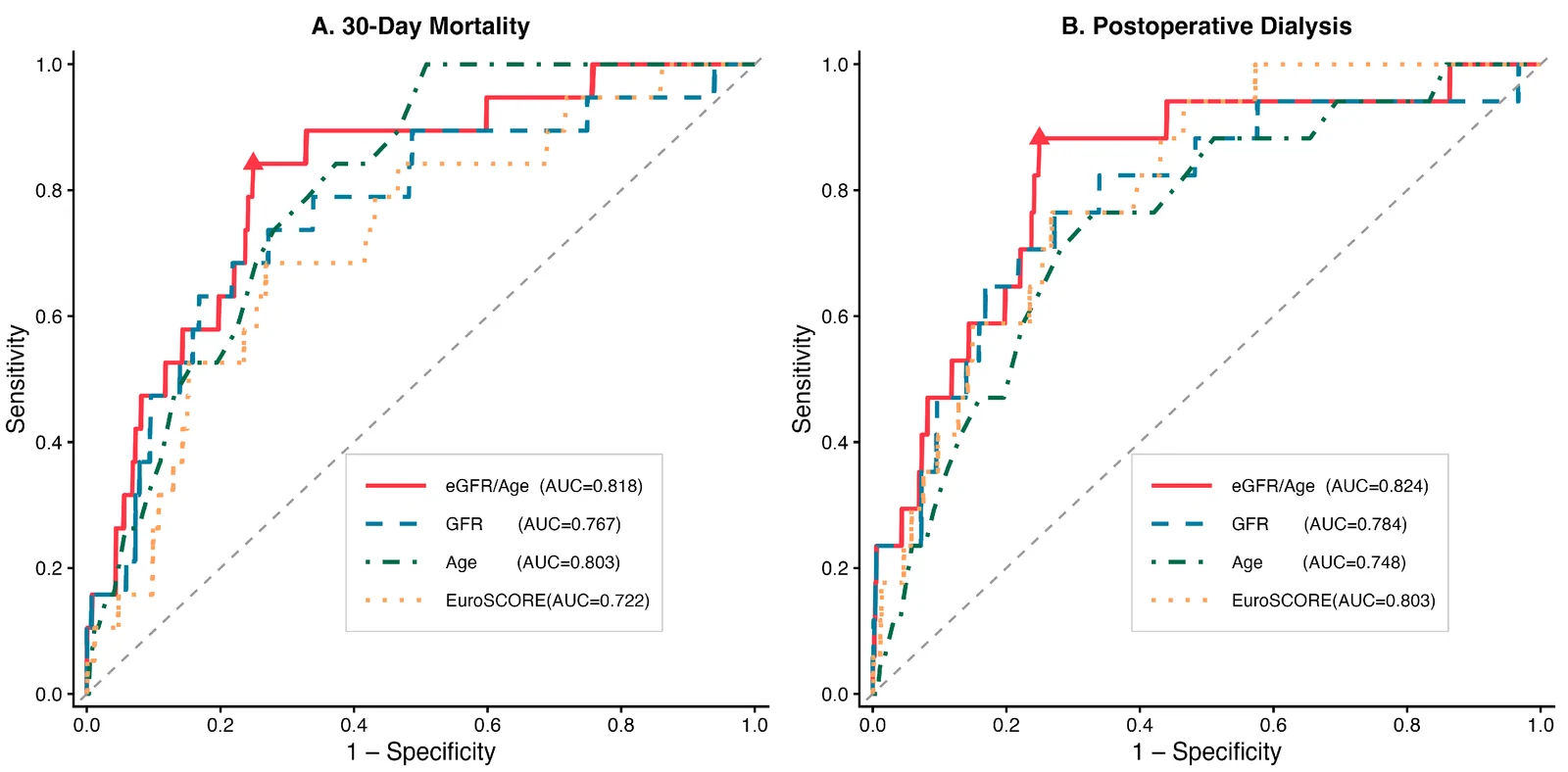
ROC curves for the eGFR-to-Age ratio and comparators for 30-day mortality (**A**) and postoperative dialysis (**B**). Red triangles indicate the outcome-specific Youden cut-offs (eGFR-to-Age ratio 1.1355 for both 30-day mortality and postoperative dialysis). AUC = area under the curve.

**Figure 2:**
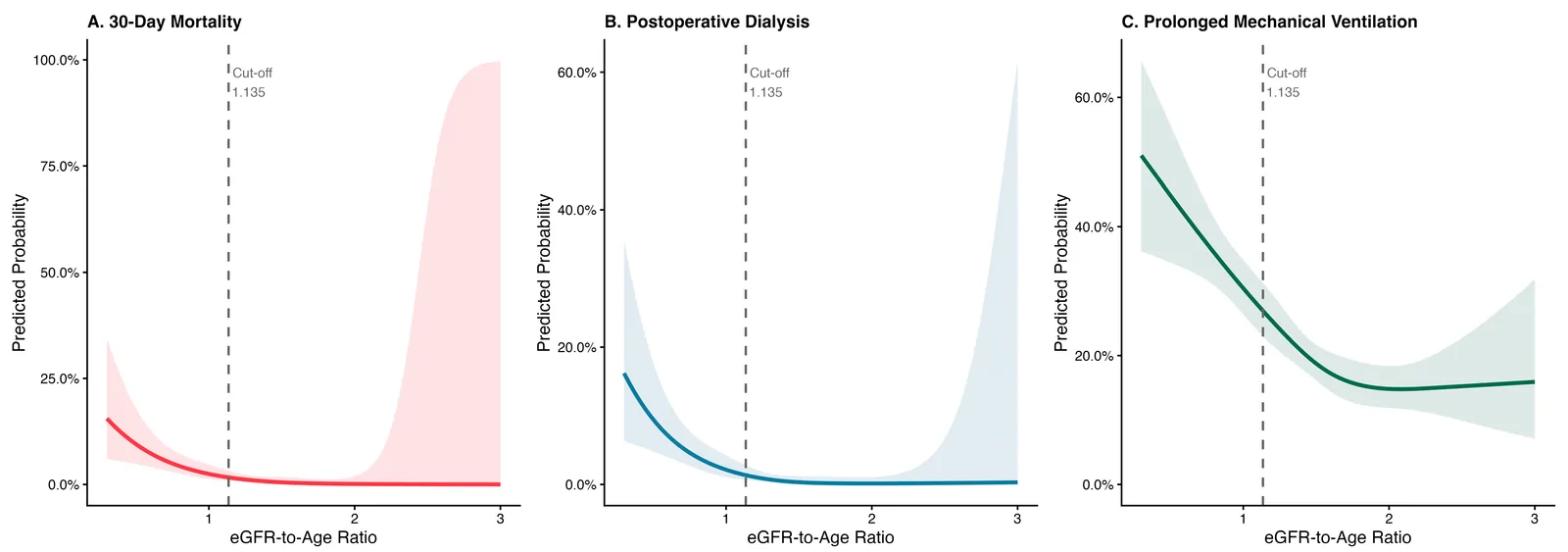
Restricted cubic spline curves depicting the association between the eGFR-to-Age ratio and predicted probability of 30-day mortality (**A**), postoperative dialysis (**B**), and prolonged mechanical ventilation (**C**). Shaded areas = 95% CI. Dashed line = the rounded 1.135 threshold used to define the groups in Table 1.

**Figure 3:**
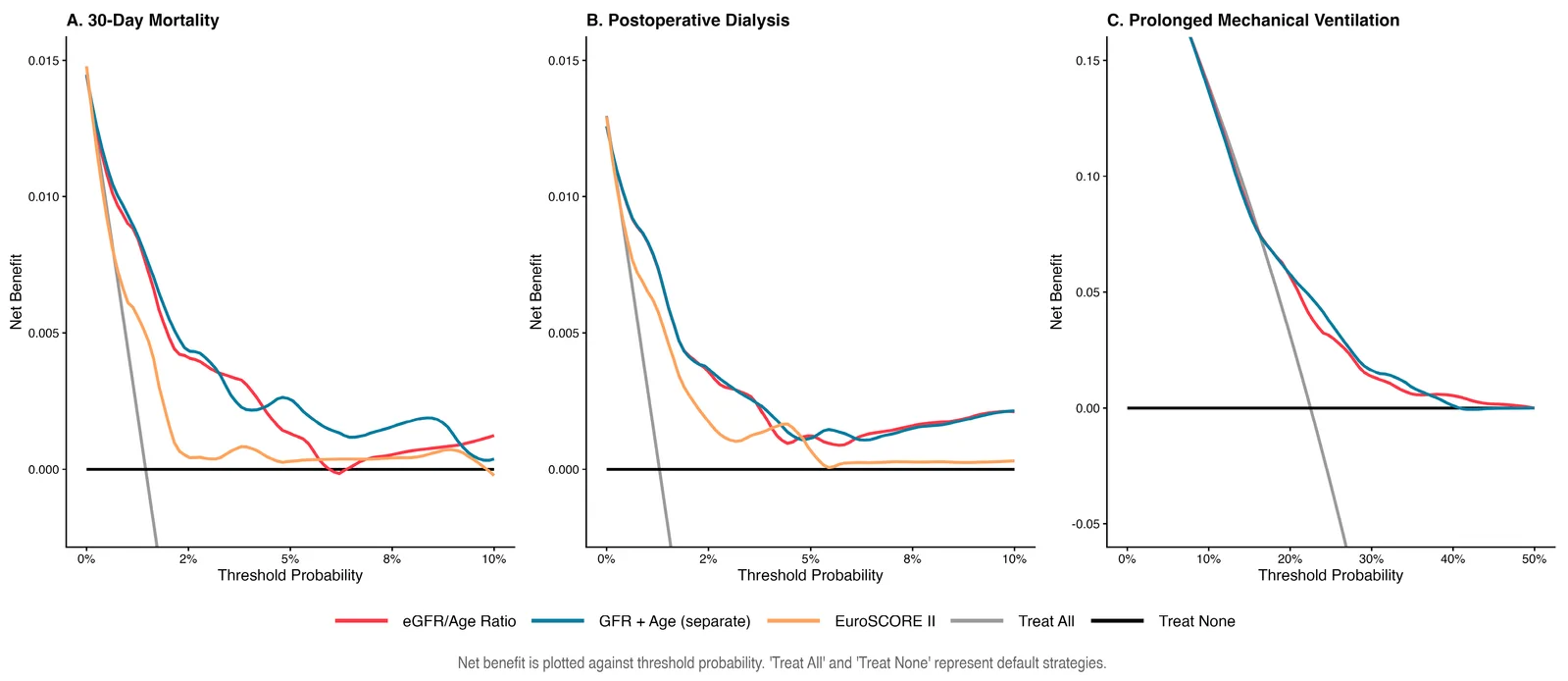
Decision curve analysis for 30-day mortality (**A**), postoperative dialysis (**B**), and prolonged mechanical ventilation (**C**). Net benefit is plotted against threshold probability. Grey and black lines represent treat-all and treat-none strategies.

**Table 1:** Baseline Characteristics of the Study Population and Comparison by eGFR-to-Age Ratio Group.

Variable	Overall (N = 1311) ^1^	Low GFR/Age (<1.135) (n = 333) ^1^	High GFR/Age (≥1.135) (n = 978) ^1^	*p*-Value ^2^
Age (years)	61.00 (54.00–67.00)	69.00 (65.00–74.00)	58.00 (52.00–63.00)	<0.001
Female sex				<0.001
Male	1053 (80.3%)	245 (73.6%)	808 (82.6%)	
Female	258 (19.7%)	88 (26.4%)	170 (17.4%)	
BMI (kg/m^2^)	27.78 (25.35–31.11)	28.08 (24.91–31.25)	27.77 (25.56–31.02)	0.799
BSA (m^2^)	1.90 (1.81–2.02)	1.87 (1.77–1.98)	1.92 (1.82–2.03)	<0.001
Hypertension	803 (61.3%)	261 (78.4%)	542 (55.4%)	<0.001
Hyperlipidemia	732 (55.8%)	108 (32.4%)	624 (63.8%)	<0.001
Diabetes mellitus	599 (45.7%)	156 (46.8%)	443 (45.3%)	0.669
Smoking status				<0.001
Never	460 (35.1%)	152 (45.6%)	308 (31.5%)	
Current	494 (37.7%)	93 (27.9%)	401 (41.0%)	
Former	357 (27.2%)	88 (26.4%)	269 (27.5%)	
COPD	96 (7.3%)	26 (7.8%)	70 (7.2%)	0.786
Preoperative dialysis	3 (0.2%)	3 (0.9%)	0 (0.0%)	0.016
NYHA class				<0.001
1	672 (51.3%)	155 (46.5%)	517 (52.9%)	
2	530 (40.4%)	162 (48.6%)	368 (37.6%)	
3	109 (8.3%)	16 (4.8%)	93 (9.5%)	
4	0 (0.0%)	0 (0.0%)	0 (0.0%)	
Ejection fraction (%)	55.00 (50.00–60.00)	55.00 (50.00–60.00)	55.00 (50.00–60.00)	0.107
Mitral regurgitation grade				<0.001
Absent	346 (26.4%)	73 (21.9%)	273 (27.9%)	
Mild	735 (56.1%)	177 (53.2%)	558 (57.1%)	
Mild-to-moderate	230 (17.5%)	83 (24.9%)	147 (15.0%)	
eGFR (mL/min/1.73 m^2^)	87.53 (73.03–99.53)	61.86 (52.70–69.14)	94.72 (84.61–101.97)	<0.001
Creatinine (mg/dL)	0.95 (0.82–1.09)	1.20 (1.06–1.36)	0.89 (0.79–1.01)	<0.001
eGFR-to-Age Ratio	1.44 (1.13–1.76)	0.92 (0.74–1.04)	1.60 (1.37–1.87)	<0.001
CRP (mg/L)	3.50 (1.62–7.28)	3.82 (1.75–8.18)	3.39 (1.58–6.95)	0.018
Albumin (g/L)	42.90 (40.60–45.00)	42.30 (39.70–44.30)	43.10 (41.10–45.30)	<0.001
HbA1c (%)	6.40 (5.80–7.90)	6.40 (5.90–7.70)	6.40 (5.80–7.90)	0.605
Hematocrit (%)	41.80 (38.50–44.70)	39.20 (35.90–42.50)	42.50 (39.50–45.20)	<0.001
WBC (×10^3^/µL)	8.19 (6.79–9.80)	7.63 (6.25–9.13)	8.39 (7.02–9.96)	<0.001
Platelet (×10^3^/µL)	232.00 (199.00–279.00)	227.00 (193.00–274.00)	234.00 (201.00–280.00)	0.030
Potassium (mEq/L)	4.45 (4.20–4.74)	4.52 (4.27–4.87)	4.43 (4.18–4.72)	<0.001
AST (U/L)	19.00 (15.00–26.00)	18.00 (15.00–26.00)	19.00 (15.00–26.00)	0.520
ALT (U/L)	19.00 (13.00–27.00)	16.00 (12.00–25.00)	19.00 (14.00–28.00)	<0.001
EuroSCORE II (%)	0.90 (0.66–1.28)	1.39 (1.05–1.96)	0.77 (0.64–1.05)	<0.001
CPB time (min)	97.00 (80.00–118.00)	96.00 (81.00–120.00)	97.00 (80.00–118.00)	0.873
Cross-clamp time (min)	52.00 (42.00–66.00)	51.00 (43.00–63.00)	53.00 (42.00–67.00)	0.228
Number of grafts				0.663
1	23 (1.8%)	5 (1.5%)	18 (1.8%)	
2	273 (20.8%)	62 (18.6%)	211 (21.6%)	
3	643 (49.0%)	177 (53.2%)	466 (47.6%)	
4	297 (22.7%)	74 (22.2%)	223 (22.8%)	
5	66 (5.0%)	13 (3.9%)	53 (5.4%)	
6	8 (0.6%)	2 (0.6%)	6 (0.6%)	
7	1 (0.1%)	0 (0.0%)	1 (0.1%)	
LIMA use	1240 (94.6%)	300 (90.1%)	940 (96.1%)	<0.001

^1^ Median (Q1–Q3); n (%). ^2^ Wilcoxon rank sum test; Pearson’s Chi-squared test; Fisher’s exact test for preoperative dialysis and number of grafts (Monte Carlo simulation) (expected cell count < 5).

**Table 2:** Postoperative Outcomes by eGFR-to-Age Ratio Group.

Variable	Overall (N = 1311) ^1^	Low GFR/Age (<1.135) (n = 333) ^1^	High GFR/Age (≥1.135) (n = 978) ^1^	*p*-Value ^2^
30-day mortality	19 (1.4%)	15 (4.5%)	4 (0.4%)	<0.001
Prolonged mechanical ventilation	295 (22.5%)	116 (34.8%)	179 (18.3%)	<0.001
Postoperative dialysis	17 (1.3%)	14 (4.2%)	3 (0.3%)	<0.001
ICU length of stay (days)	2.00 (1.00–3.00)	3.00 (1.00–5.00)	2.00 (1.00–3.00)	<0.001
Hospital length of stay (days)	7.00 (6.00–11.00)	9.00 (7.00–14.00)	7.00 (6.00–10.00)	<0.001
Acute kidney injury	276 (21.1%)	140 (42.0%)	136 (13.9%)	<0.001
Postoperative atrial fibrillation	245 (18.7%)	95 (28.5%)	150 (15.3%)	<0.001
Reintubation	61 (4.7%)	28 (8.4%)	33 (3.4%)	<0.001
Bleeding revision	51 (3.9%)	12 (3.6%)	39 (4.0%)	0.882
Pleural effusion	97 (7.4%)	35 (10.5%)	62 (6.3%)	0.017
IABP requirement	16 (1.2%)	5 (1.5%)	11 (1.1%)	0.570

^1^ Median (Q1–Q3); n (%). ^2^ Wilcoxon rank sum test; Pearson’s Chi-squared test; Fisher’s exact test for 30-day mortality, postoperative dialysis, and IABP requirement (expected cell count < 5). The three patients receiving preoperative dialysis were coded as not having AKI and were excluded from the AKI regression analyses.

**Table 3:** Multivariable Regression Analysis for Primary and Secondary Outcomes.

Outcome	Variable	OR/β	95% CI	*p*-Value	Model AUC
30-Day Mortality	★ eGFR-to-Age Ratio	0.786	0.679–0.915	0.001	0.858
	Age (years)	1.051	0.979–1.136	0.187	
	Ejection fraction (%)	0.954	0.908–1.005	0.067	
	LIMA use	0.215	0.077–0.677	0.005	
Postoperative Dialysis	★ eGFR-to-Age Ratio	0.735	0.633–0.851	<0.001	0.869
	Age (years)	1.003	0.935–1.081	0.936	
	Ejection fraction (%)	0.926	0.879–0.976	0.004	
	LIMA use	0.227	0.073–0.818	0.014	
Acute Kidney Injury ^‡^	★ eGFR-to-Age Ratio	0.822	0.783–0.863	<0.001	0.691
	Age (years)	0.974	0.951–0.998	0.038	
	Ejection fraction (%)	0.987	0.971–1.003	0.113	
	LIMA use	1.231	0.688–2.318	0.501	
Prolonged Mechanical Ventilation ^†^	★ eGFR-to-Age Ratio	0.953	0.911–0.998	0.041	0.676
	Age (years)	1.026	1.002–1.051	0.037	
	Ejection fraction (%)	0.990	0.974–1.006	0.196	
	CPB time (min)	1.013	1.009–1.017	<0.001	
	Albumin (g/L)	0.947	0.913–0.983	0.004	

OR = Odds Ratio; CI = Confidence Interval; ★ = primary predictor of interest; all models adjusted for age to assess independent contribution of eGFR-to-Age Ratio. Odds ratios for the eGFR-to-Age ratio are expressed per 0.1-unit increase. ^‡^ Secondary outcome; patients receiving preoperative dialysis were excluded (n = 1308; 276 events). ^†^ Prolonged mechanical ventilation was analyzed as a secondary exploratory outcome; CPB time was included as an intraoperative covariate given its established association with postoperative ventilatory failure.

**Table 4:** Comparison of Discriminatory Performance: GFR-to-Age Ratio vs. Alternative Predictors.

Outcome	Predictor	AUC	95% CI	*p* vs. GFR/Age
30-Day Mortality	eGFR-to-Age Ratio	0.818	0.726–0.909	-
	eGFR + Age (separate)	0.831	0.742–0.920	0.195
	eGFR alone	0.767	0.649–0.885	0.005
	Age alone	0.803	0.730–0.876	0.716
	EuroSCORE II	0.722	0.608–0.837	0.018
Postoperative Dialysis	eGFR-to-Age Ratio	0.824	0.722–0.926	-
	eGFR + Age (separate)	0.822	0.720–0.924	0.480
	eGFR alone	0.784	0.662–0.906	0.022
	Age alone	0.748	0.635–0.862	0.215
	EuroSCORE II	0.803	0.718–0.889	0.649
Acute Kidney Injury ^†^	eGFR-to-Age Ratio	0.686	0.648–0.724	-
	eGFR + Age (separate)	0.690	0.652–0.728	0.436
	eGFR alone	0.689	0.651–0.727	0.705
	Age alone	0.619	0.581–0.657	<0.001
	EuroSCORE II	0.645	0.607–0.683	0.016
Prolonged Mechanical Ventilation	eGFR-to-Age Ratio	0.620	0.583–0.657	-
	eGFR + Age (separate)	0.628	0.591–0.665	0.119
	eGFR alone	0.589	0.551–0.627	<0.001
	Age alone	0.621	0.583–0.659	0.928
	EuroSCORE II	0.621	0.584–0.658	0.966

DeLong method for pairwise ROC comparison; AUC = Area Under the Curve; CI = Confidence Interval. ^†^ Patients receiving preoperative dialysis were excluded (n = 1308).

**Table 5:** Diagnostic Performance of eGFR-to-Age Ratio Models at Outcome-Specific Cut-offs.

Measure	30-Day Mortality	Postoperative Dialysis	Prolonged Mechanical Ventilation
Predicted probability cut-off ^†^	0.016	0.014	0.209
Sensitivity (95% CI)	84.2% (59.5–95.8%)	88.2% (62.3–97.9%)	71.9% (66.3–76.8%)
Specificity (95% CI)	75.1% (72.6–77.4%)	75.0% (72.6–77.4%)	48.8% (45.7–51.9%)
PPV (95% CI)	4.7% (2.8–7.7%)	4.4% (2.6–7.4%)	29.0% (25.7–32.4%)
NPV (95% CI)	99.7% (99.0–99.9%)	99.8% (99.2–100%)	85.7% (82.5–88.4%)
LR+	3.38	3.53	1.40
LR−	0.210	0.157	0.576
Hosmer–Lemeshow *p* value	0.149	0.135	0.032

^†^ Predicted-probability cut-offs were selected using the Youden index separately for each outcome. The corresponding eGFR-to-Age ratio cut-offs were 1.1355 for 30-day mortality and postoperative dialysis and 1.5110 for prolonged mechanical ventilation. Table 1 uses the rounded threshold of 1.135 to define its groups; therefore, its group counts should not be used to calculate the diagnostic performance shown here. PPV = positive predictive value; NPV = negative predictive value; LR+ = positive likelihood ratio; LR− = negative likelihood ratio.

## Data Availability

The data supporting the findings of this study are available from the corresponding author upon reasonable request. The data are not publicly available due to privacy and ethical restrictions.

## Supplementary Materials

The following supporting information can be downloaded at: https://www.mdpi.com/article/10.3390/biomedicines14102386/s1, Figure S1. ICU (A) and hospital (B) length of stay by eGFR-to-Age ratio group. Diamond = median. Mann–Whitney U test. Figure S2. Forest plot of univariable odds ratios for the association between the eGFR-to-Age ratio and 30-day mortality by prespecified subgroups. Odds ratios are expressed per 0.1-unit increase in the eGFR-to-Age ratio. Log scale; dashed line = OR of 1.0. Figure S3. Calibration plots for 30-day mortality (A), postoperative dialysis (B), and prolonged mechanical ventilation (C). Points = risk groups; error bars = 95% CI; dashed line = perfect calibration. Table S1. Univariable Analysis of GFR-to-Age Ratio for Each Outcome. Table S2. Variance inflation factors and linearity assessment of continuous covariates in the multivariable models. Table S3. Sensitivity analysis with eGFR and age entered as separate continuous terms. Table S4. Observed event rates and model-predicted probabilities across eGFR-to-Age ratio values. Table S5. Comparison of the eGFR-to-Age ratio with alternative functional forms. Table S6. Firth penalized regression and sensitivity analysis excluding patients receiving preoperative dialysis. Table S7. Univariable associations of all candidate predictors with each outcome. Table S8. Full multivariable model equations (regression coefficients and intercepts). Table S9. Comparison of linear, restricted cubic spline, and piecewise linear models for acute kidney injury. TRIPOD checklist.

## Abbreviations

The following abbreviations are used in this manuscript:

AKI	Acute kidney injury
ALT	Alanine aminotransferase
AST	Aspartate aminotransferase
AUC	Area under the curve
BMI	Body mass index
BSA	Body surface area
CABG	Coronary artery bypass grafting
CI	Confidence interval
CKD-EPI	Chronic Kidney Disease Epidemiology Collaboration
COPD	Chronic obstructive pulmonary disease
CPB	Cardiopulmonary bypass
CRP	C-reactive protein
eGFR	Estimated glomerular filtration rate
EuroSCORE II	European System for Cardiac Operative Risk Evaluation II
GFR	Glomerular filtration rate
HbA1c	Glycated hemoglobin
IABP	Intra-aortic balloon pump
ICU	Intensive care unit
IQR	Interquartile range
KDIGO	Kidney Disease: Improving Global Outcomes
LIMA	Left internal mammary artery
LR+	Positive likelihood ratio
LR−	Negative likelihood ratio
MV	Mechanical ventilation
NPV	Negative predictive value
NYHA	New York Heart Association
OR	Odds ratio
PPV	Positive predictive value
ROC	Receiver operating characteristic
VIF	Variance inflation factor
WBC	White blood cell count
