# Supplementary material for: Age-Normalized Baseline Renal Function: A Simple Risk Marker in Coronary Artery Bypass Grafting
Source: Biomedicines. 2026 Oct 19;14(10):2386. doi: 10.3390/biomedicines14102386 (PMC13701741; doi:10.3390/biomedicines14102386)
Supplement: Supplementary file 1 [file biomedicines-14-02386-s001.zip › biomedicines-4619141-supplementary.pdf]

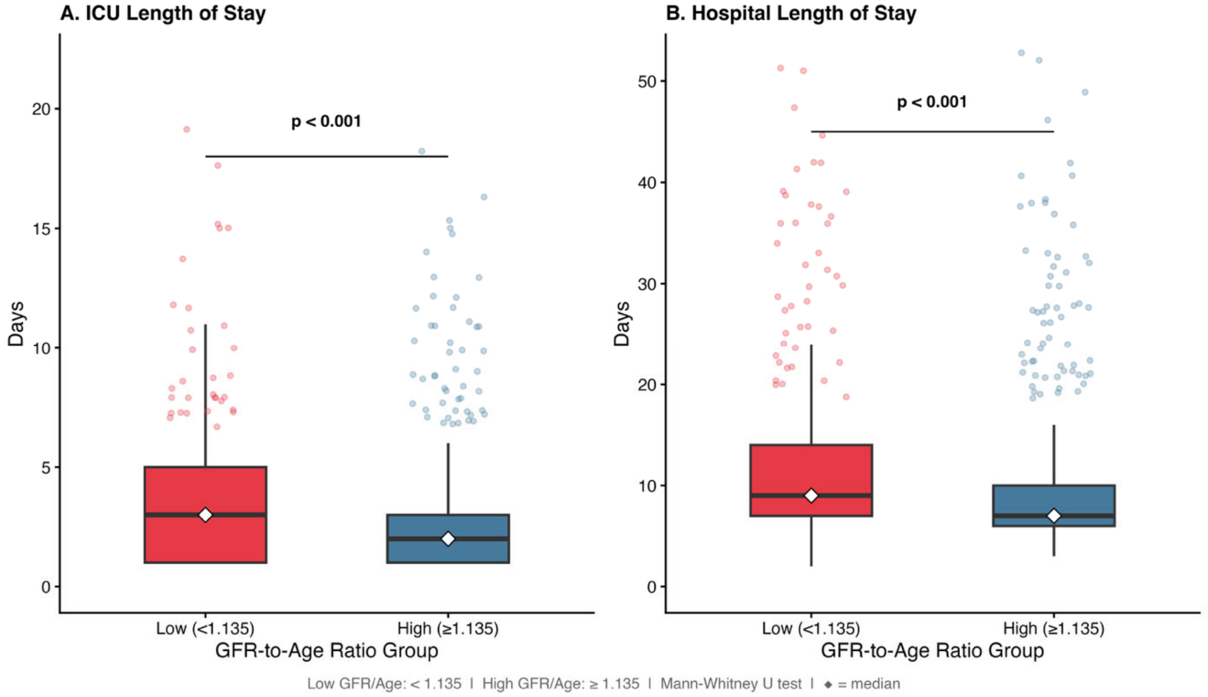

Figure S1. ICU (A) and hospital (B) length of stay by eGFR-to-Age ratio group. Diamond = median. Mann-Whitney U test.

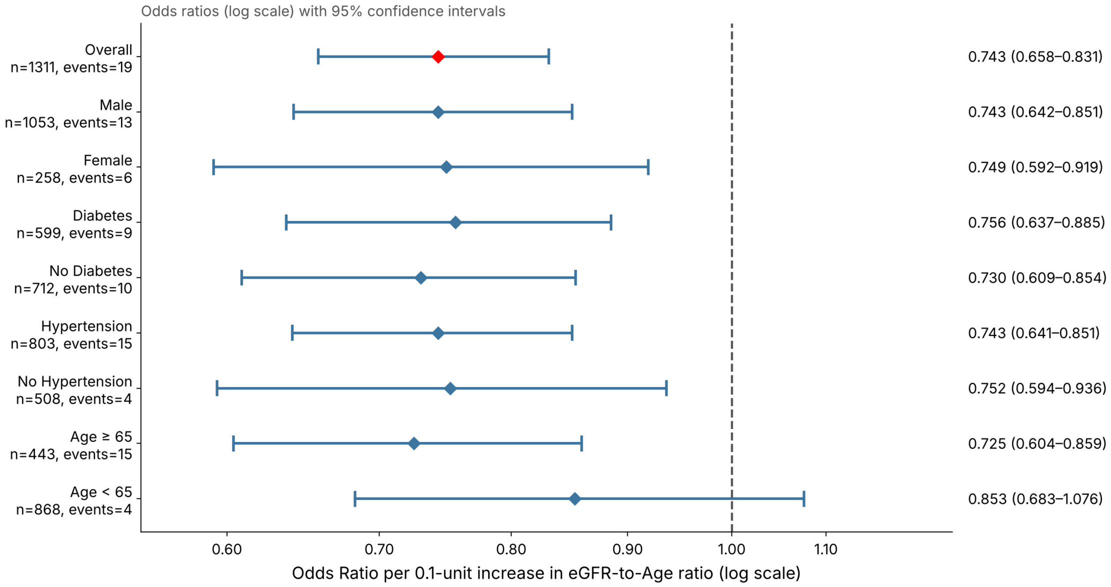

Figure S2. Forest plot of univariable odds ratios for the association between the eGFR-to-Age ratio and 30-day mortality by prespecified subgroups. Odds ratios are expressed per 0.1-unit increase in the eGFR-to-Age ratio. Log scale; dashed line = OR of 1.0.

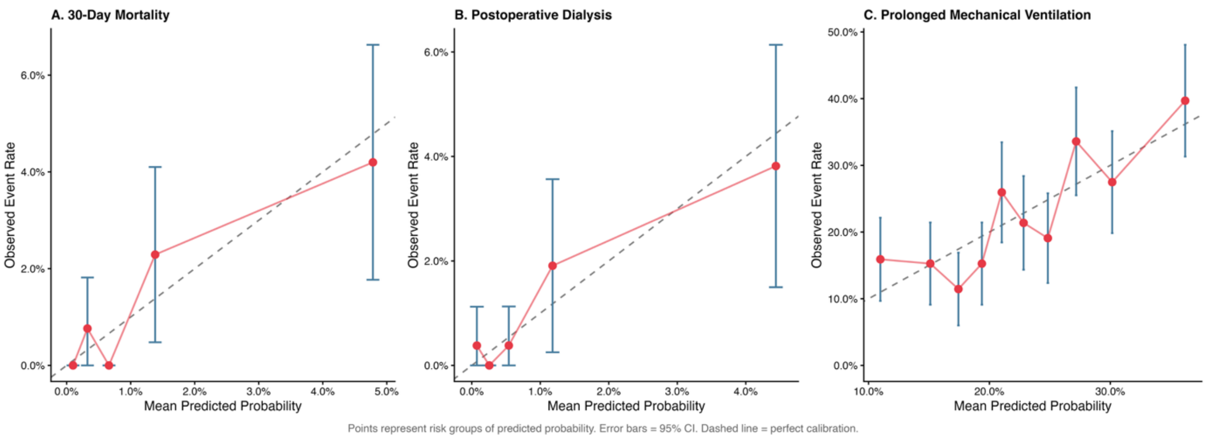

Figure S3. Calibration plots for 30-day mortality (A), postoperative dialysis (B), and prolonged mechanical ventilation (C). Points = risk groups; error bars = 95% CI; dashed line = perfect calibration.

Table S1. Univariable Analysis of GFR-to-Age Ratio for Each Outcome

Outcome	OR / β	95% CI	p-value	AUC
**30-Day Mortality (OR)**	0.743	0.658 – 0.831	<0.001	0.818
**Prolonged Mechanical Ventilation (OR)**	0.914	0.887 – 0.940	<0.001	0.62
**Postoperative Dialysis (OR)**	0.731	0.642 – 0.824	<0.001	0.824
**Acute Kidney Injury (OR)**	0.855	0.826 – 0.883	<0.001	0.686
**ICU Length of Stay — days (β)**	-1.780	-2.348 – -1.212	<0.001	—
**Hospital Length of Stay — days (β)**	-3.580	-4.791 – -2.368	<0.001	—

OR = Odds Ratio; β = regression coefficient; CI = Confidence Interval; AUC = Area Under the Curve. ORs are expressed per 0.1-unit increase in the eGFR-to-Age ratio; β coefficients per 1-unit increase. The AKI analysis excludes the three patients receiving preoperative dialysis (n=1,308).

Table S2. Variance Inflation Factors and Linearity Assessment of Continuous Covariates in the Multivariable Models

Outcome / Model	Covariate	VIF	p for non-linearity
30-Day Mortality	eGFR-to-Age Ratio	2.84	0.933
	Age (years)	2.86	0.121
	Ejection fraction (%)	1.00	0.634
	LIMA use	1.03	—

Outcome / Model	Covariate	VIF	p for non-linearity
Postoperative Dialysis	eGFR-to-Age Ratio	2.84	0.625
	Age (years)	2.86	0.902
	Ejection fraction (%)	1.00	0.480
	LIMA use	1.03	—
Prolonged Mechanical Ventilation	eGFR-to-Age Ratio	2.86	0.030
	Age (years)	2.84	0.004
	Ejection fraction (%)	1.02	0.880
	CPB time (min)	1.00	0.656
	Albumin (g/L)	1.04	0.001
Acute Kidney Injury	eGFR-to-Age Ratio	2.92	<0.001
	Age (years)	2.94	0.009
	Ejection fraction (%)	1.00	0.906
	LIMA use	1.03	—

VIF = variance inflation factor; LIMA = left internal mammary artery; CPB = cardiopulmonary bypass. Non-linearity was tested for each continuous covariate within its multivariable model (Table 3) by likelihood-ratio comparison of a restricted cubic spline (4 knots; 3 knots for ejection fraction because of tied values) with a linear term, all other covariates being entered linearly. — = not applicable (binary variable). The AKI model excludes the three patients receiving preoperative dialysis (n=1,308).

Table S3. Sensitivity Analysis with eGFR and Age Entered as Separate Continuous Terms

Outcome	Variable	OR	95% CI	p-value	Model AUC
30-Day Mortality	eGFR (per mL/min/1.73 m^2)	0.965	0.943–0.988	0.002	0.855
	Age (per year)	1.090	1.023–1.168	0.010	
	Ejection fraction (%)	0.954	0.908–1.006	0.069	
	LIMA use	0.212	0.075–0.672	0.005	
Postoperative Dialysis	eGFR (per mL/min/1.73 m^2)	0.954	0.931–0.977	<0.001	0.864
	Age (per year)	1.050	0.986–1.124	0.141	
	Ejection fraction (%)	0.926	0.878–0.976	0.004	
	LIMA use	0.224	0.072–0.818	0.014	
Prolonged Mechanical Ventilation	eGFR (per mL/min/1.73 m^2)	0.990	0.983–0.998	0.011	0.679

Outcome	Variable	OR	95% CI	p-value	Model AUC
	Age (per year)	1.035	1.018–1.054	<0.001	
	Ejection fraction (%)	0.990	0.974–1.006	0.196	
	CPB time (min)	1.013	1.009–1.017	<0.001	
	Albumin (g/L)	0.949	0.914–0.985	0.005	
Acute Kidney Injury	eGFR (per mL/min/1.73 m^2)	0.965	0.957–0.973	<0.001	0.692
	Age (per year)	1.014	0.996–1.032	0.137	
	Ejection fraction (%)	0.987	0.971–1.003	0.104	
	LIMA use	1.264	0.699–2.407	0.455	

Models are identical to those in Table 3 except that the eGFR-to-Age ratio was replaced by eGFR and age entered as separate continuous terms. AUCs of the corresponding eGFR-to-Age ratio models (Table 3): 0.858 (30-day mortality), 0.869 (postoperative dialysis), and 0.676 (prolonged mechanical ventilation). OR = odds ratio; CI = confidence interval (profile likelihood); AUC = area under the curve; LIMA = left internal mammary artery; CPB = cardiopulmonary bypass. AKI: patients receiving preoperative dialysis excluded (n=1,308); AUC of the corresponding eGFR-to-Age ratio model 0.691.

Table S4. Observed Event Rates and Model-Predicted Probabilities Across eGFR-to-Age Ratio Values

eGFR-to-Age ratio	n	30-Day Mortality	Postoperative Dialysis	Acute Kidney Injury*
**A. Observed event rates, n (%)**				
<0.8	103	8 (7.8)	7 (6.8)	59/100 (59.0)
0.8 to <1.0	120	3 (2.5)	3 (2.5)	53 (44.2)
1.0 to <1.2	175	5 (2.9)	5 (2.9)	37 (21.1)
1.2 to <1.4	213	1 (0.5)	1 (0.5)	28 (13.1)
1.4 to <1.6	224	1 (0.4)	0 (0.0)	36 (16.1)
1.6 to <1.8	173	1 (0.6)	0 (0.0)	26 (15.0)
≥1.8	303	0 (0.0)	1 (0.3)	37 (12.2)
**B. Model-predicted probability, %**				
0.6		7.4	6.9	—
0.8		4.2	3.8	—
1.0		2.4	2.1	—
1.2		1.3	1.1	—
1.4		0.7	0.6	—
1.6		0.4	0.3	—
1.8		0.2	0.2	—

eGFR-to-Age ratio	n	30-Day Mortality	Postoperative Dialysis	Acute Kidney Injury*
2.0		0.1	0.1	—

Panel A shows observed event rates by eGFR-to-Age ratio band; panel B shows predicted probabilities from univariable logistic regression models of 30-day mortality and postoperative dialysis on the eGFR-to-Age ratio at representative ratio values. *AKI excludes the three patients receiving preoperative dialysis (all in the <0.8 band; denominator 100 in that band). Model-predicted AKI probabilities are not shown because the association between the ratio and AKI departed from linearity (Supplementary Table S2); observed AKI rates are shown in panel A.

Table S5. Comparison of the eGFR-to-Age Ratio with Alternative Functional Forms

Outcome	Predictor	AUC (95% CI)	p vs ratio
30-Day Mortality	eGFR-to-Age ratio	0.818 (0.726–0.909)	Reference
	Deviation from age-expected eGFR	0.663 (0.517–0.809)	<0.001
	eGFR + age + eGFR × age	0.831 (0.745–0.917)	0.223
Postoperative Dialysis	eGFR-to-Age ratio	0.824 (0.722–0.926)	Reference
	Deviation from age-expected eGFR	0.696 (0.545–0.848)	0.005
	eGFR + age + eGFR × age	0.826 (0.724–0.928)	0.768
Acute Kidney Injury*	eGFR-to-Age ratio	0.686 (0.648–0.724)	Reference
	Deviation from age-expected eGFR	0.659 (0.621–0.698)	0.109
	eGFR + age + eGFR × age	0.688 (0.650–0.726)	0.769
Prolonged Mechanical Ventilation	eGFR-to-Age ratio	0.620 (0.583–0.657)	Reference
	Deviation from age-expected eGFR	0.531 (0.491–0.571)	<0.001
	eGFR + age + eGFR × age	0.627 (0.590–0.665)	0.177

Deviation from age-expected eGFR = observed eGFR minus eGFR predicted from age by linear regression in this cohort (predicted eGFR = 146.65 − 1.025 × age). p values for the eGFR × age interaction term: 0.724 (30-day mortality), 0.286 (postoperative dialysis), 0.015 (AKI), and 0.803 (prolonged mechanical ventilation). AUC comparisons by the DeLong method. *Patients receiving preoperative dialysis excluded (n=1,308).

Table S6. Firth Penalized Regression and Sensitivity Analysis Excluding Patients Receiving Preoperative Dialysis

Analysis	Variable	OR	95% CI	p-value
**A. Firth penalized logistic regression**				
30-Day Mortality	eGFR-to-Age ratio (per 0.1)	0.789	0.684–0.915	0.002
	Age (years)	1.047	0.975–1.130	0.212

Analysis	Variable	OR	95% CI	p-value
	Ejection fraction (%)	0.954	0.909–1.004	0.069
	LIMA use	0.212	0.078–0.639	0.007
Postoperative Dialysis	eGFR-to-Age ratio (per 0.1)	0.740	0.641–0.854	<0.001
	Age (years)	1.000	0.933–1.075	0.992
	Ejection fraction (%)	0.928	0.882–0.976	0.005
	LIMA use	0.221	0.074–0.752	0.018
**B. Postoperative dialysis excluding patients receiving preoperative dialysis (n=1,308; 17 events)**				
Postoperative Dialysis	eGFR-to-Age ratio (per 0.1)	0.715	0.609–0.836	<0.001
	Age (years)	0.993	0.924–1.072	0.856
	Ejection fraction (%)	0.930	0.882–0.980	0.006
	LIMA use	0.208	0.066–0.758	0.010

Panel A: same covariates as in Table 3, fitted with Firth penalized likelihood; 95% CIs and p values from the profile penalized likelihood. Panel B: standard maximum-likelihood logistic regression (profile-likelihood 95% CIs); model AUC 0.871. None of the three patients receiving preoperative dialysis was classified as having postoperative dialysis. OR = odds ratio; CI = confidence interval; LIMA = left internal mammary artery.

Table S7. Univariable Associations of All Candidate Predictors with Each Outcome

Predictor	30-Day Mortality	Postoperative Dialysis	Acute Kidney Injury*	Prolonged Mechanical Ventilation
eGFR-to-Age ratio (per 0.1)	0.743 (0.658–0.831); p<0.001	0.731 (0.642–0.824); p<0.001	0.855 (0.826–0.883); p<0.001	0.914 (0.887–0.940); p<0.001
eGFR (per mL/min/1.73 m²)	0.954 (0.936–0.972); p<0.001	0.949 (0.930–0.968); p<0.001	0.962 (0.955–0.969); p<0.001	0.982 (0.976–0.989); p<0.001
Age (per year)	1.145 (1.080–1.221); p<0.001	1.111 (1.048–1.183); p<0.001	1.050 (1.034–1.067); p<0.001	1.048 (1.032–1.064); p<0.001
Ejection fraction (per %)	0.953 (0.911–1.000); p=0.042	0.930 (0.887–0.976); p=0.003	0.985 (0.970–1.000); p=0.048	0.985 (0.971–1.000); p=0.052
LIMA use	0.115 (0.044–0.335); p<0.001	0.129 (0.046–0.415); p<0.001	0.897 (0.517–1.643); p=0.711	0.421 (0.258–0.698); p<0.001
CPB time (per min)	1.005 (0.991–1.019); p=0.451	1.008 (0.992–1.021); p=0.291	1.005 (1.001–1.009); p=0.018	1.012 (1.008–1.017); p<0.001
Albumin (per g/L)	0.887 (0.800–0.992); p=0.028	0.858 (0.772–0.962); p=0.006	0.952 (0.918–0.986); p=0.006	0.935 (0.903–0.968); p<0.001

Predictor	30-Day Mortality	Postoperative Dialysis	Acute Kidney Injury*	Prolonged Mechanical Ventilation

Values are odds ratios (95% profile-likelihood confidence intervals) and p values from univariable logistic regression. *Patients receiving preoperative dialysis excluded (n=1,308). LIMA = left internal mammary artery; CPB = cardiopulmonary bypass.

Table S8. Full Multivariable Model Equations (Regression Coefficients and Intercepts)

Outcome	Term	Coefficient (log-odds)	Standard error
30-Day Mortality	Intercept	-0.9985	3.3834
	eGFR-to-Age ratio (per 0.1)	-0.2403	0.0754
	Age (years)	0.0501	0.0380
	Ejection fraction (%)	-0.0471	0.0257
	LIMA use (1 = yes)	-1.5351	0.5469
Postoperative Dialysis	Intercept	4.1332	3.2163
	eGFR-to-Age ratio (per 0.1)	-0.3081	0.0746
	Age (years)	0.0029	0.0367
	Ejection fraction (%)	-0.0764	0.0264
	LIMA use (1 = yes)	-1.4829	0.6043
Acute Kidney Injury*	Intercept	3.4559	1.1726
	eGFR-to-Age ratio (per 0.1)	-0.1955	0.0249
	Age (years)	-0.0259	0.0125
	Ejection fraction (%)	-0.0131	0.0083
	LIMA use (1 = yes)	0.2077	0.3083
Prolonged Mechanical Ventilation	Intercept	-0.6316	1.3285
	eGFR-to-Age ratio (per 0.1)	-0.0478	0.0234
	Age (years)	0.0256	0.0123
	Ejection fraction (%)	-0.0105	0.0081
	CPB time (min)	0.0128	0.0022
	Albumin (g/L)	-0.0540	0.0189

Predicted probability = 1 / [1 + exp(−LP)], where LP = intercept + Σ(coefficient × value); the eGFR-to-Age ratio term is entered as (ratio × 10), i.e., per 0.1-unit. These are the models presented in Table 3 of the main text. *Patients receiving preoperative dialysis excluded (n=1,308).

Table S9. Comparison of Linear, Restricted Cubic Spline, and Piecewise Linear Models for Acute Kidney Injury

Model (ratio term)	Ratio range	OR per 0.1-unit increase (95% CI)	AUC	AIC	LR test vs linear
Linear	Entire range	0.822 (0.783–0.863)	0.691	1250.0	Reference
Restricted cubic spline (4 knots)	Entire range	Non-linear	0.691	1229.6	p<0.001
Piecewise linear (knot at 1.2)	<1.2	0.679 (0.619–0.743)	0.692	1224.8	p<0.001
	≥1.2	0.925 (0.867–0.986)			

All models were adjusted for age, ejection fraction, and LIMA use and exclude the three patients receiving preoperative dialysis (n=1,308; 276 events). Spline knots were placed at the 5th, 35th, 65th, and 95th percentiles of the ratio (0.717, 1.269, 1.619, and 2.302). The piecewise knot at 1.2 was chosen post hoc on the basis of observed event rates (Supplementary Table S4); the difference between the two piecewise slopes was significant (p<0.001). AIC = Akaike information criterion; LR = likelihood ratio; AUC = area under the curve; LIMA = left internal mammary artery.

TRIPOD Checklist

Section/Topic	Item	Checklist item	Reported in (section)
**Title and abstract**			
Title	1	Identify the study as developing and/or validating a multivariable prediction model, the target population, and the outcome to be predicted.	Title
Abstract	2	Provide a summary of objectives, study design, setting, participants, sample size, predictors, outcome, statistical analysis, results, and conclusions.	Abstract
**Introduction**			
Background and objectives	3a	Explain the medical context and rationale for developing or validating the model, including references to existing models.	Introduction, paragraphs 1–3
	3b	Specify the objectives, including whether the study describes the development or validation of the model or both.	Introduction, last paragraph (including intended use)
**Methods**			
Source of data	4a	Describe the study design or source of data (e.g., randomized trial, cohort, or registry data).	Methods, Study Design and Patient Population (retrospective single-center cohort)
	4b	Specify the key study dates, including start of accrual, end of accrual, and, if applicable, end of follow-up.	Methods, Study Design and Patient Population (February 2025–February 2026; 30-day follow-up)
Participants	5a	Specify key elements of the study setting including number and location of centres.	Methods, Study Design and Patient Population
	5b	Describe eligibility criteria for participants.	Methods, Study Design and Patient Population
	5c	Give details of treatments received, if relevant.	Methods (isolated CABG); Table 1 (operative characteristics)
Outcome	6a	Clearly define the outcome that is predicted by the prediction model, including how and when assessed.	Methods, Outcome Definitions (postoperative dialysis, AKI, prolonged mechanical ventilation); method of ascertainment of 30-day mortality not reported
	6b	Report any actions to blind assessment of the outcome to be predicted.	Not applicable (retrospective analysis of routinely recorded outcomes); not reported
Predictors	7a	Clearly define all predictors used in developing or validating the multivariable prediction model, including how and when they were measured.	Methods, Statistical Analysis (eGFR equation, IDMS-standardized creatinine, covariates)
	7b	Report any actions to blind assessment of predictors for the outcome and other predictors.	Not applicable (preoperative predictors recorded before outcome occurrence); not reported
Sample size	8	Explain how the study size was arrived at.	Methods, Study Design (all consecutive eligible patients) and Statistical Analysis (events per variable)

Section/Topic	Item	Checklist item	Reported in (section)
Missing data	9	Describe how missing data were handled with details of any imputation method.	Methods, Study Design (complete-case analysis; patients with missing data for required variables excluded; number excluded not reported)
Statistical analysis methods	10a	Describe how predictors were handled in the analyses.	Methods, Statistical Analysis (continuous terms, linearity assessment, per 0.1-unit scaling)
	10b	Specify type of model, all model-building procedures (including any predictor selection), and method for internal validation.	Methods, Statistical Analysis (a priori covariates, logistic and Firth regression, bootstrap validation)
	10d	Specify all measures used to assess model performance and, if relevant, to compare multiple models.	Methods, Statistical Analysis (AUC, DeLong, Hosmer–Lemeshow, calibration slope and intercept, decision curve analysis)
Risk groups	11	Provide details on how risk groups were created, if done.	Methods, Statistical Analysis (Youden-derived cut-offs, consolidated threshold statement)
**Results**			
Participants	13a	Describe the flow of participants through the study, including the number of participants with and without the outcome and, if applicable, a summary of the follow-up time.	Results, Baseline Characteristics; Table 2 (outcome events). Number of excluded patients and loss to follow-up not reported
	13b	Describe the characteristics of the participants, including the number of participants with missing data for predictors and outcome.	Results; Table 1
Model development	14a	Specify the number of participants and outcome events in each analysis.	Results; Tables 3 and 4; Supplementary Tables S1–S6
	14b	If done, report the unadjusted association between each candidate predictor and outcome.	Supplementary Tables S1 and S7 (all candidate predictors)
Model specification	15a	Present the full prediction model to allow predictions for individuals (i.e., all regression coefficients, and model intercept or baseline survival at a given time point).	Table 3 (odds ratios); Supplementary Table S8 (full model equations: coefficients and intercepts)
	15b	Explain how to use the prediction model.	Supplementary Table S8 (model equations); Supplementary Table S4 (risk table)
Model performance	16	Report performance measures (with CIs) for the prediction model.	Results; Tables 3–5; Internal Validation
**Discussion**			
Limitations	18	Discuss any limitations of the study (such as nonrepresentative sample, few events per predictor, missing data).	Discussion, limitations paragraph
Interpretation	19b	Give an overall interpretation of the results, considering objectives, limitations, and results from similar studies, and other relevant evidence.	Discussion

Section/Topic	Item	Checklist item	Reported in (section)
Implications	20	Discuss the potential clinical use of the model and implications for future research.	Discussion; Conclusions
**Other information**			
Supplementary information	21	Provide information about the availability of supplementary resources, such as study protocol, Web calculator, and data sets.	Supplementary Materials; Data Availability Statement
Funding	22	Give the source of funding and the role of the funders for the present study.	Funding statement

TRIPOD checklist for prediction model development with internal validation. Items applicable only to external validation studies (10c, 10e, 12, 13c, 17, 19a) are not applicable and are omitted.
